# Construction and validation of a machine learning-based prediction model for venous thromboembolism in lung transplant recipients supported by ECMO

**DOI:** 10.3389/fmed.2026.1808657

**Published:** 2026-06-04

**Authors:** Yan Zhu, Fei Zeng, Mei-Juan Lan, Jiang-Shu-Yuan Liang, Ling-Yun Cai, Pei-Pei Gu, Lu-Yao Guo

**Affiliations:** Department of Nursing, The Second Affiliated Hospital of Zhejiang University School of Medicine, Hangzhou, China

**Keywords:** ECMO, lung transplant, machine learning-based prediction model, risk prediction, venous thromboembolism

## Abstract

**Introduction:**

This study investigated risk factors for venous thromboembolism (VTE) in lung transplant recipients receiving ECMO and developed a VTE risk prediction model based on machine learning (ML).

**Methods:**

We retrospectively reviewed the medical records of 189 patients who underwent elective lung transplantation at the Second Affiliated Hospital of Zhejiang University from May 2023 to November 2024. Recursive Feature Elimination was used to analyze risk factors for VTE during ECMO after lung transplantation. Six ML models were established. Grid search combined with 5-fold cross-validation identified optimal parameters for the models. The models’ predictive performance was assessed using 5-fold cross-validation. The evaluation metrics included accuracy, sensitivity, specificity, positive predictive value (PPV), negative predictive value (NPV), receiver operating characteristic curve (ROC) and its area under the curve (AUC), as well as the F1 score. Six predictive factors were included in the model construction.

**Results:**

The Random Forest model performed best. The area under the ROC of the model on the validation set was 0.895 (95% CI: 0.788–1.000); the accuracy was 89.7%, sensitivity 89.7%, specificity 89.5%, PPV 0.946%, and negative predictive value 81.0%. The calibration curve showed strong agreement between predicted probabilities and observed outcomes; decision curve analysis indicated significant clinical utility across relevant threshold probabilities.

**Conclusion:**

The ML-derived VTE risk prediction model for lung transplant patients showed strong predictive ability and clinical utility confirmed by decision curve analysis.

## 1 Introduction

Lung transplantation is the ultimate treatment for end-stage lung diseases and has saved countless lives. Extracorporeal membrane oxygenation (ECMO) is an extracorporeal life support technology that plays a pivotal role in the management of cardiopulmonary failure (1). ECMO not only provides preoperative stabilization and postoperative circulatory and respiratory assistance, but it can also be proactively used during surgery to ensure adequate oxygen supply, stabilize hemodynamics, and secure safe passage through the transplant process (2). ECMO thus significantly enhances the overall efficacy of lung transplantation, and the clinical outcomes of patients supported by ECMO therapy have continued to improve (3, 4).

While ECMO offers critical life support, it is accompanied by substantial risks of severe complications (5). An analysis of data from 2,885 adult patients supported by ECMO reported by the Extracorporeal Life Support Organization identified bleeding, thrombosis, infection, and hemolysis as the most common causes of ECMO failure (6). These complications can arise throughout the entire period of support, with thrombotic events being particularly prevalent, occurring at a rate of nearly 36% across various sites (7). Both catheter-related and systemic thrombosis pose significant risks during ECMO support and after decannulation, directly impacting patient prognosis (8). The mortality rate associated with these complications is approximately 40%, making these complications critical determinants of ECMO treatment success or failure (9). Currently, there is a lack of validated VTE risk prediction models and an absence of disease-specific assessment tools for lung transplant recipients, impeding the development of evidence-based thromboprophylaxis strategies.

ML offers superior capabilities in handling large-scale, multi-dimensional datasets, enabling accurate identification of risk factors and the generation of robust predictive models (10, 11). In this study, we developed a validated, interpretable VTE risk model for lung transplant patients using ML. This model provides clinicians with a visualized tool to guide thromboprophylaxis.

## 2 Methods

### 2.1 Study patients

This study was approved by the Institutional Review Board of the Second Affiliated Hospital of Zhejiang University School of Medicine (Approval No.: IRB-2024-1498). All methods were performed in accordance with the relevant guidelines and regulations. All participants were fully informed and consented to participate in this study. The internal validation dataset included data derived from the medical records of patients who underwent lung transplantation at the Second Affiliated Hospital of Zhejiang University School of Medicine, China between May 2023 to May 2024. The temporal validation dataset included data obtained from the medical records of patients who underwent elective lung transplantation at the same institution between June 2024 and November 2024, representing a distinct temporal cohort to assess model performance stability over time.

The study inclusion criteria were as follows: (1) age ≥18 years and provided informed consent; (2) patients undergoing elective lung transplantation; (3) patients receiving ECMO support during the postoperative period; and (4) patients who underwent bilateral lower limb deep vein color Doppler ultrasound (CUS) examination between the postoperative period and discharge. The study exclusion criteria were as follows: (1) patients with incomplete case data; (2) patients with hematologic comorbidities or coagulation dysfunction; and (3) patients with severe contraindications to ECMO.

### 2.2 ECMO management protocol

When ECMO is employed after lung transplantation, the anticoagulation regimen should be individualized based on the patient’s specific characteristics, the ECMO configuration, and the institution’s experience. In our center, routine anticoagulation is achieved with unfractionated heparin, and anticoagulation monitoring primarily relies on the activated clotting time (ACT), targeting a range of 160–180 s. Activated partial thromboplastin time (APTT) may be used adjunctively, with a target approximately 1.5–2.5 times the normal value (roughly 50–70 s). For patients with active bleeding, heparin should be discontinued immediately; in cases of minor bleeding, the anticoagulation target can be lowered or the heparin dose modestly reduced.

### 2.3 Diagnosis of VTE

The examination was performed on-site in the ICU by a trained vascular ultrasound technologist. The initial assessment was conducted within 48 h post-operation using bedside CUS, while a nurse simultaneously drew blood for D-dimer measurement in the laboratory. The diagnosis of deep vein thrombosis (DVT) was confirmed by a vascular surgeon or thrombosis specialist based on multiple ultrasonographic criteria—including incompressibility, intraluminal echogenicity, absence of color Doppler flow, and abnormal pulse-wave Doppler spectral patterns—integrated with the vascular anatomical location and the clinical context (12). If new clinical symptoms (e.g., limb swelling, pain) or an elevated D-dimer level emerged, a repeat examination was performed immediately; for negative results in high-risk patients, a follow-up scan was required after 5–7 days.

### 2.4 Data processing

Patient data were sourced from the hospital’s comprehensive electronic medical record system, resulting in minimal missing values. Variables with a missing rate exceeding 10% were excluded. For the remaining features, no missing values were present in the dataset; thus, no imputation was performed. To standardize the data, all categorical variables were transformed into binary vectors using one-hot encoding; the data types of these variables were converted into indicator variables (0 for absence/nonexistence or 1 for presence/existence). Given the imbalance in the included data, oversampling was performed during the modeling process. The SMOTE algorithm was used to oversample the minority class (patients with venous thrombosis) in the training dataset.

### 2.5 Variable processing

Data were retrospectively collected from the electronic medical record system of the hospital. A total of 22 features were included, comprising demographic data, preoperative data (including comorbidities), postoperative imaging and laboratory test results (including coagulation function indicators, complete blood count, and biochemical parameters), and medication use (including anticoagulant administration). We employed Recursive Feature Elimination to identify the most critical predictors of postoperative venous thrombosis from 22 initial candidate features (12). Dimensionality reduction of the relevant variables was achieved through feature selection, which in turn enhanced the performance and interpretability of the model.

### 2.6 Development and verification of the ML models

ML was conducted using Python 3.7.9, and the SHAP 0.42.1 package was installed to perform visualization analysis. We used six distinct ML algorithms to construct risk prediction models: (i) Logistic Regression (LR) serves as a classical parametric baseline with strong interpretability; (ii) Support Vector Machine (SVM) is effective in handling high-dimensional data via kernel-based margin maximization; (iii) K-Nearest Neighbors (KNN) provides a non-parametric instance-based learning perspective; (iv) Random Forest (RF), (v) eXtreme Gradient Boosting (XG Boost), and (vi) Adaptive Boosting (AdaBoost) represent three distinct ensemble strategies—bagging, gradient boosting, and adaptive boosting, respectively—allowing us to evaluate how different ensemble mechanisms perform on this specific clinical dataset (13).

Grid search was employed to systematically enumerate all possible combinations of predefined hyperparameter values across a specified search space for each algorithm. The primary objective was threefold: (i) to identify the optimal hyperparameter configuration that maximizes model performance on the training data; (ii) to ensure an objective and reproducible parameter selection process devoid of subjective bias; and (iii) to guarantee fair comparison among the six models by applying a consistent optimization strategy. In each iteration, 5-fold cross-validation was used to evaluate the performance of each hyperparameter combination, with the combination yielding the highest mean cross-validated AUC selected as the final configuration. The complete grid search space for each algorithm is provided in Supplementary material S1.

**Figure 1:**
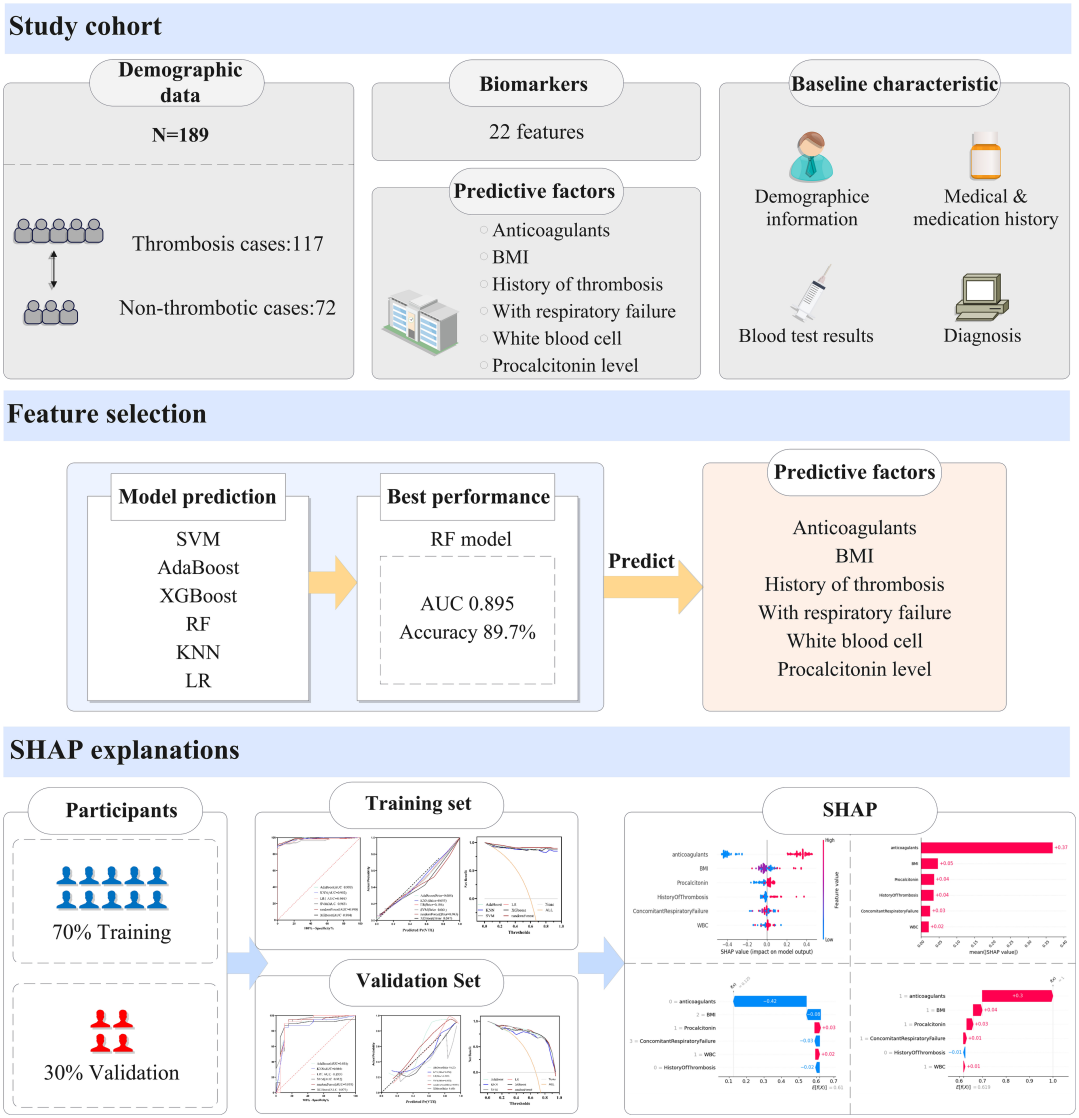
Machine learning pipeline. The figure graphically summarizes the machine learning steps.

Performance metrics included accuracy, sensitivity, specificity, PPV, NPV, and the area under the ROC curve. The mathematical equations for all comparison metrics are provided in Supplementary material S2. The SHAP algorithm was applied to evaluate the contribution of each feature to the final model output, thereby enhancing model interpretability. The overall study flow is illustrated in Figure 1.

### 2.7 Statistical analysis

Count data are expressed as frequencies (percentages), and inter-group comparisons were conducted using the χ^2^ test. Differences with *p* < 0.05 were regarded as statistically significant.

## 3 Results

### 3.1 Patient demographics

A total of 189 patients were enrolled, including 117 postoperative DVT cases and 72 patients without postoperative DVT. Among them, 152 were male (80.42%) and 37 were female (19.58%). No significant differences were observed in baseline characteristics between the VTE and non-VTE groups. The clinical profiles of the VTE and non-VTE cohorts are summarized in Table 1.

**Table 1:** Demographic and clinical characteristics.

Variable		Total	Non-thrombotic	Thrombotic	χ^2^	*P*
Gender	Male	152(80.42%)	60(83.33%)	92(78.63%)	0.626	0.429
	Female	37(19.58%)	12(16.67%)	25(21.37%)		
Diagnosis	ILD	93(49.21%)	43(59.72%)	50(42.74%)	7.074	0.29
	COPD	66(34.92%)	23(31.94%)	43(36.75%)		
	Other/Mixed	30(15.87%)	6(8.33%)	24(20.51%)		
Age(years)	≤60	106(56.08%)	43(59.72%)	63(53.85%)	0.625	0.429
	>60	83(43.92%)	29(40.28%)	54(46.15%)		
History of hypertension	No	144(76.19%)	54(75%)	90(76.92%)	0.091	0.763
	Yes	45(23.81%)	18(25%)	27(23.08%)		
History of diabetes	No	141(74.6%)	51(70.83%)	90(76.92%)	0.872	0.350
	Yes	48(25.4%)	21(29.17%)	27(23.08%)		
History of tumor	No	157(83.07%)	59(81.94%)	98(83.76%)	0.105	0.746
	Yes	32(16.93%)	13(18.06%)	19(16.24%)		
History of coronary heart disease	No	154(81.48%)	56(77.78%)	98(83.76%)	1.057	0.304
	Yes	35(18.52%)	16(22.22%)	19(16.24%)		
6MWD	<200 [200,300] >300	120(63.49%) 53(28.04%) 16(8.47%)	47(65.28%) 18(25%) 7(9.72%)	73(62.39%) 35(29.91%) 9(7.69%)	0.659	0.719
FEV₁/FVC%	<30% [30,50%] [50,70%]	124(65.61%) 62(32.8%) 3(1.59%)	47(65.28%) 24(33.33%) 1(1.39%)	77(65.81%) 38(32.48%) 2(1.71%)	0.041	0.98
FVC%	<35% [35,50%] [50,80%]	110(58.2%) 74(39.15%) 5(2.65%)	44(61.11%) 26(36.11%) 2(2.78%)	66(56.41%) 48(41.03%) 3(2.56%)	0.452	0.798
FEV₁% predicted	≤35% [35,50%] [50,80%]	71(37.57%) 115(60.85%) 3(1.59%)	28(38.89%) 43(59.72%) 1(1.39%)	43(36.75%) 72(61.54%) 2(1.71%)	0.107	0.948
Pre-transplant respiratory support modalities	Oxygen therapy	169(89.42%)	5(2.65%)	106(90.6%)	2.219	0.330
	Invasive ventilation	15(7.94%)	8(11.11%)	7(5.98%)		
	ECMO support	5(2.65%)	1(1.39%)	4(3.42%)		

ILD = Interstitial Lung Disease, COPD = Chronic Obstructive Pulmonary Disease, 6MWD = 6-Minute Walking Distance, FEV₁ = forced expiratory volume in one second, FVC = forced vital capacity, Other/Mixed includes non-obstructive, non-restrictive diagnoses and patients with multifactorial, overlapping, or idiopathic pulmonary conditions.

### 3.2 Feature selection via the recursive feature elimination

Recursive Feature Elimination was employed to identify risk factors closely associated with thrombosis. Using the criteria of minimal feature count and maximal predictive accuracy, six variables exerting the greatest influence on VTE were selected: anticoagulant therapy, body mass index (BMI), history of thrombosis, procalcitonin, concomitant respiratory failure, and white-blood-cell count. These are illustrated in Supplementary Table 1.

### 3.3 Evaluation of the performance of the ML models for predicting DVT in ECMO-supported patients

**Figure 2:**
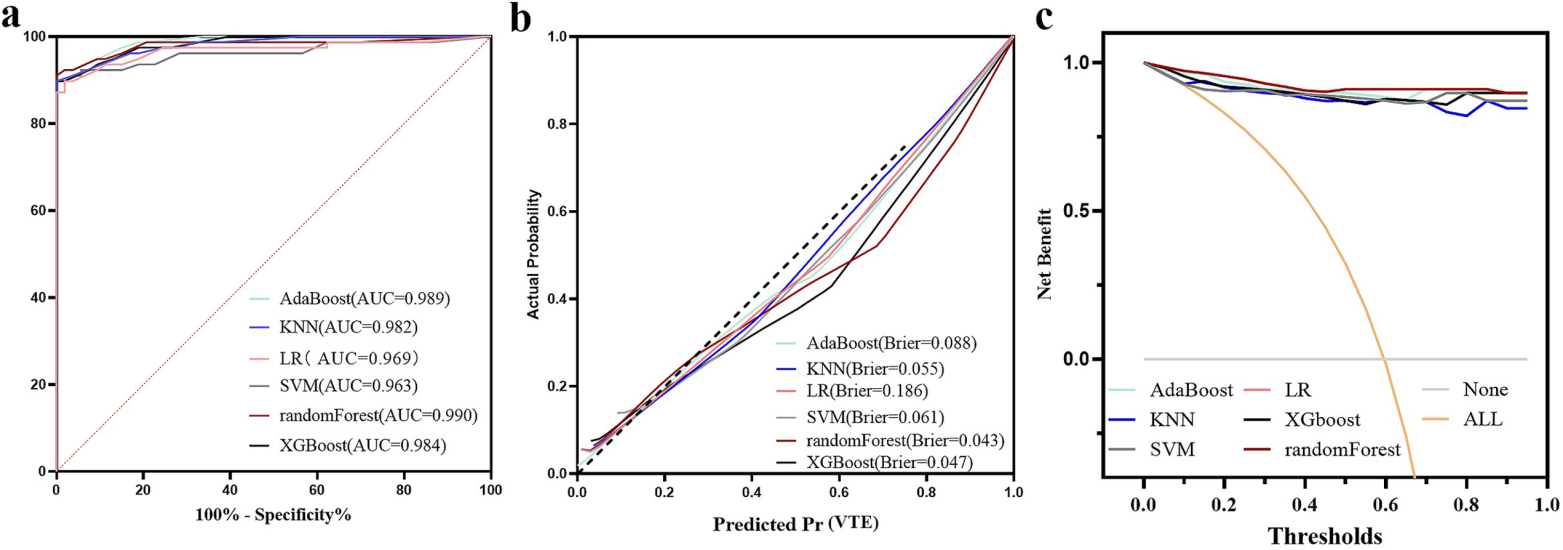
Comparison of the performance of six algorithms on the training set: **(a)** The first graph represents the ROC curve; **(b)** The second graph represents the calibration curve; **(c)** The third graph represents the DCA decision curve. AUC = area under curve, KNN = *K*-nearest neighbors, SVM = support vector machine, LR = logistic regression, VTE = venous thromboembolism.

**Figure 3:**
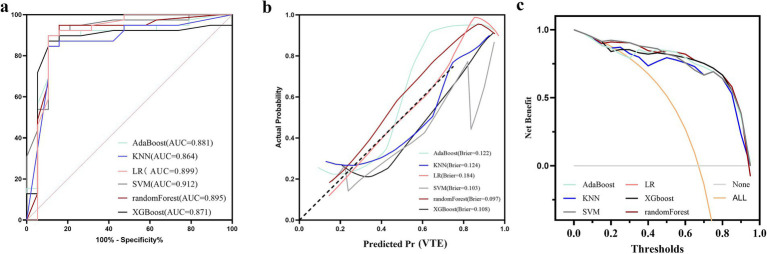
Comparison of the performance of six algorithms on the test set. **(a)** The first figure represents the ROC curve. **(b)** The second figure represents the calibration curve. **(c)** The third figure represents the DCA decision curve. AUC = area under curve, KNN = *K*-nearest neighbors, SVM = support vector machine, LR = logistic regression, VTE = venous thromboembolism.

In the six machine-learning models evaluated for DVT prediction, the RF model achieved the best performance. Consequently, we selected the RF model for subsequent temporal validation. The relevant results are presented in Figures 2, 3 and Tables 2, 3.

**Table 2:** Model prediction performance in the training set.

Algorithm	AUC (95%CI)	Accuracy	Sensitivity	Specificity	PPV	NPV	F1 Score
SVM	0.963 (0.930–0.996)	0.924	0.872	1.000	1.000	0.841	0.932
Adaboost	0.989 (0.978–1.000)	0.947	0.910	1.000	1.000	0.833	0.953
XGBoost	0.984 (0.968–0.999)	0.931	0.897	0.981	0.986	0.867	0.940
RF	0.990 (0.959–1.000)	0.947	0.910	1.000	1.000	0.883	0.953
KNN	0.982 (0.964–0.9993)	0.924	0.897	0.962	0.972	0.864	0.933
LR	0.969 (0.939–0.999)	0.664	0.987	0.189	0.642	0.909	0.778

AUC = Area Under Curve, SVM = Support Vector Machine, AdaBoost = Adaptive Boosting, XGBoost = eXtreme Gradient Boosting, RF = Random Forest, KNN = K-Nearest Neighbors, LR = Logistic Regression, PPV=Positive Predictive Value, NPV=Negative Predictive Value.

**Table 3:** Model prediction performance in the validation set.

Algorithm	AUC (95%CI)	Accuracy	Sensitivity	Specificity	PPV	NPV	F1 Score
SVM	0.912 (0.827–0.998)	0.845	0.846	0.842	0.917	0.727	0.880
Adaboost	0.881 (0.777–0.986)	0.897	0.897	0.895	0.946	0.810	0.921
XGBoost	0.871 (0.760–0.982)	0.879	0.872	0.895	0.944	0.773	0.907
RF	0.895 (0.788–1.000)	0.897	0.897	0.895	0.946	0.810	0.921
KNN	0.864 (0.755–0.973)	0.862	0.872	0.842	0.919	0.762	0.895
LR	0.899 (0.788–1.000)	0.776	1.000	0.316	0.750	1.000	0.857

AUC = Area Under Curve, SVM = Support Vector Machine, AdaBoost = Adaptive Boosting, XGBoost = eXtreme Gradient Boosting, RF = Random Forest, KNN = K-Nearest Neighbors, LR = Logistic Regression, PPV=Positive Predictive Value, NPV=Negative Predictive Value.

### 3.4 Important prediction characteristics and model interpretability

**Figure 4:**
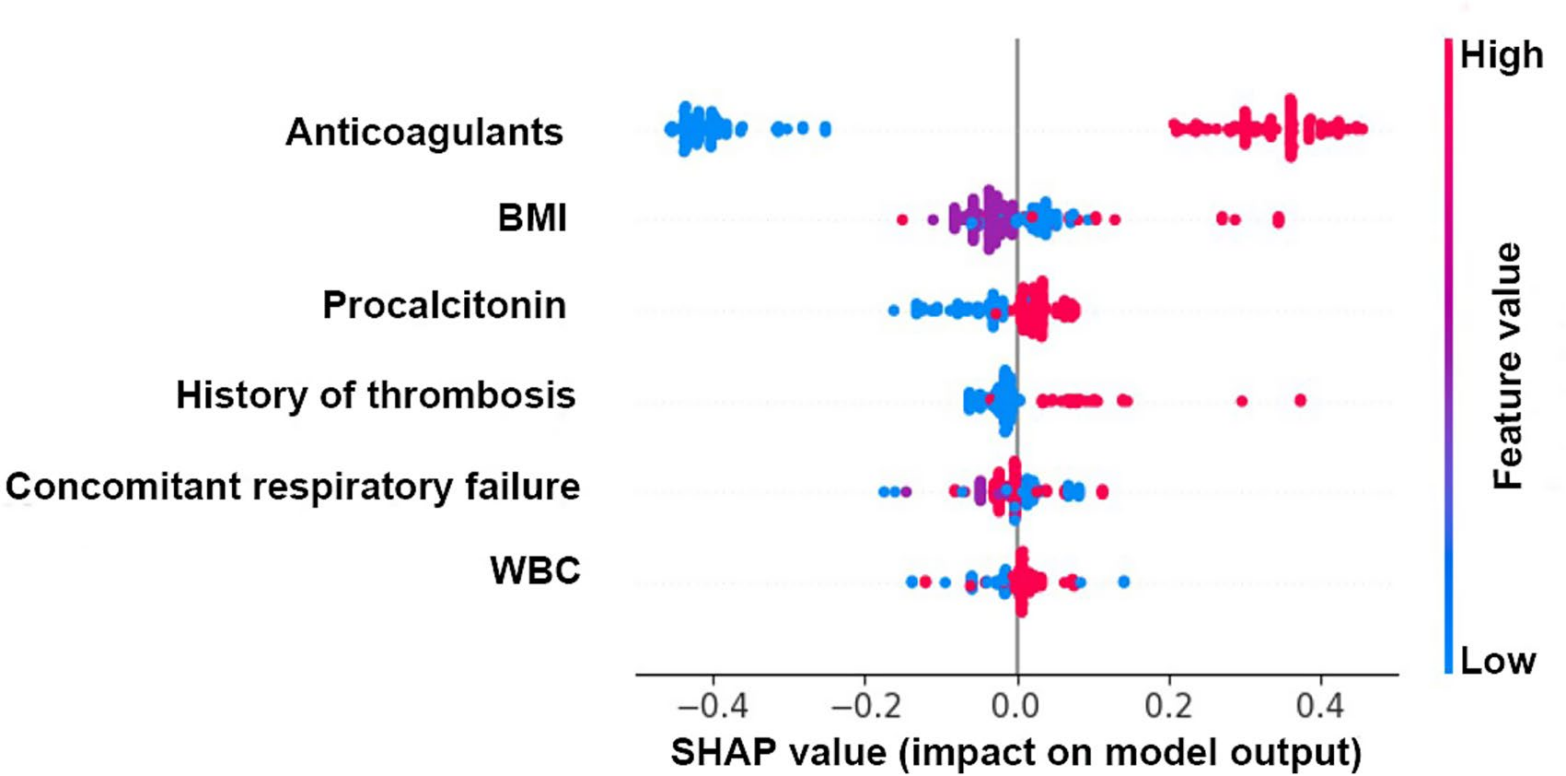
A scatter plot of SHAP values. This plot is utilized to elucidate the influence of individual features on the model output within the RF model. The *x*-axis represents the SHAP values, which quantify both the magnitude and direction of the impact of each feature on the model’s prediction outcomes. The *y*-axis lists the names of the various features. Each point in the figure corresponds to a single sample, with the color of the point indicating the magnitude of the feature value—red signifies a high feature value, while blue denotes a low feature value. The position of the point along the *x*-axis reflects the direction and extent of the feature’s influence on the model output. BMI = Body Mass Index, WBC = White Blood Cell.

**Figure 5:**
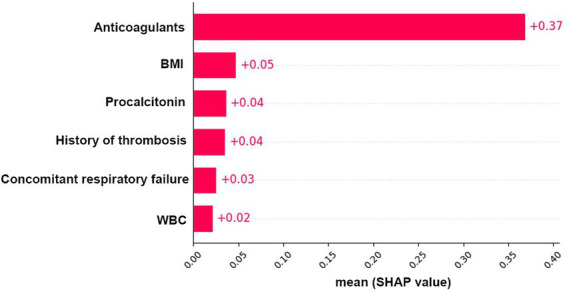
The mean absolute SHAP values (mean(|SHAP value|)) for each feature in the RF model are calculated. These values are utilized to quantify the average degree of influence that each feature exerts on the model’s predictive outcomes. Based on the analysis of the mean SHAP values illustrated in the preceding figure, the average importance of the features with respect to the output of the RF model is ranked. BMI = Body Mass Index, WBC = White Blood Cell.

**Figure 6:**
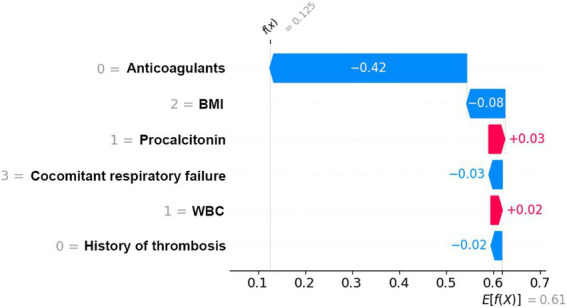
The SHAP waterfall plot for patients at low risk of thrombosis. A waterfall plot illustrates the impact of different variables on a function *f*(*x*), as well as the cumulative effect of these impacts. Each bar in the plot represents the contribution of a variable to the increase or decrease in the function value. BMI = Body Mass Index, WBC = White Blood Cell.

**Figure 7:**
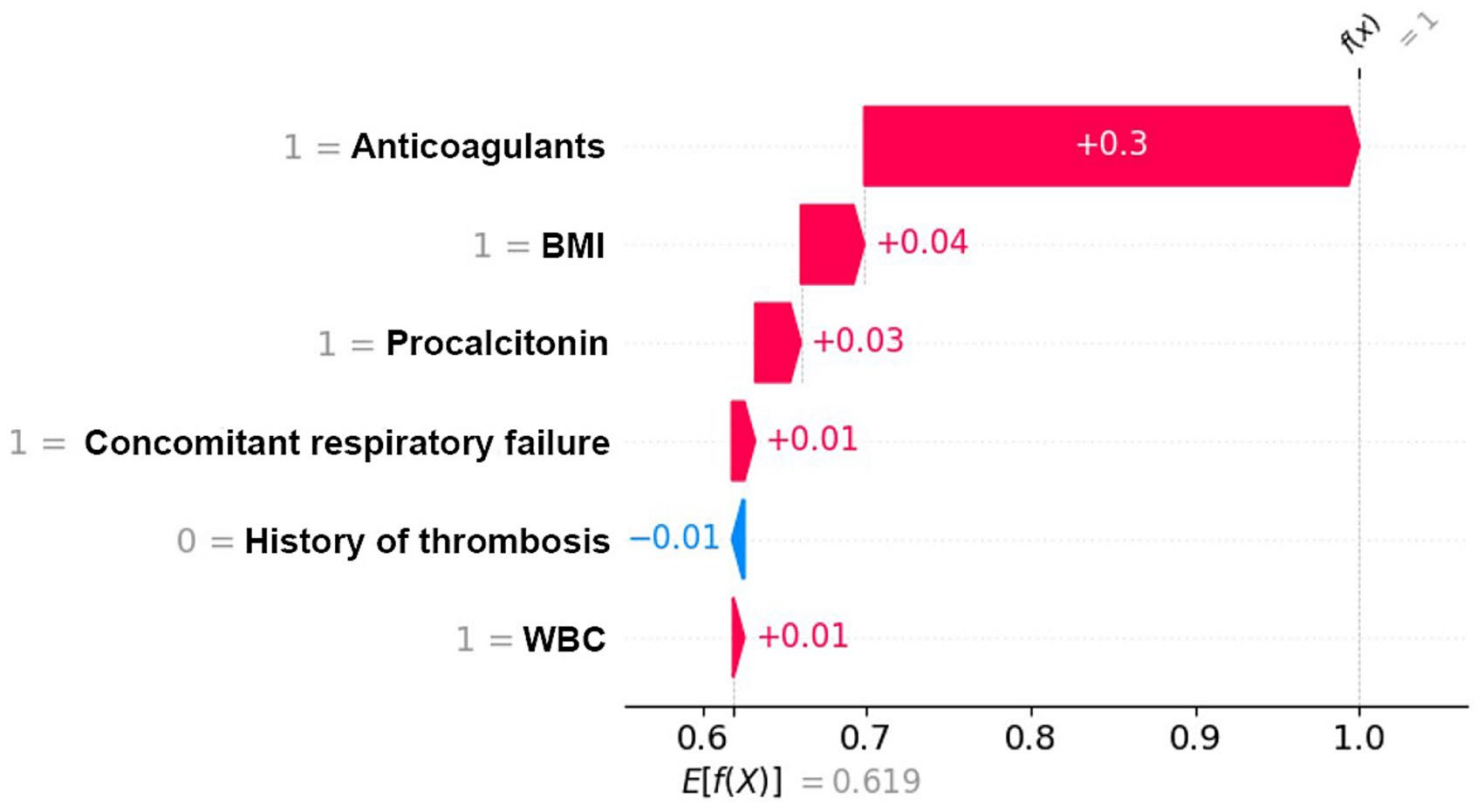
The SHAP waterfall plot for patients at high risk of recurrence. A waterfall plot is employed to depict the influence of various variables on a function *f(x)*, as well as the cumulative effect of these influences. Each bar in the plot represents the contribution of a variable to the increase or decrease in the function value. BMI = Body Mass Index WBC = White Blood Cell.

The SHAP algorithm was used to provide a visual interpretation of the RF model. This algorithm quantified the influence of each feature on the prediction outcomes and ranked the importance of six predictive factors in the following order: anticoagulant drugs, BMI, history of thrombosis, procalcitonin, concomitant respiratory failure, and white blood cells in Figures 4, 5. Two case examples are shown in Figures 6, 7: one with a low risk, characterized by a low SHAP score (0.125), and another with a high risk, characterized by a high SHAP score (1). The contribution of each feature to the samples is illustrated in the figures.

### 3.5 Comparison between random forest and the Caprini risk score

The random forest model demonstrates a significant advantage over the traditional Caprini risk score (CRS) in predicting deep-vein thrombosis risk, especially in terms of predictive accuracy and sensitivity in Table 4.

**Table 4:** Caprini risk assessment model predictive model.

Model	Accuracy	AUC (95%CI)	Thrombosis Criteria	Sensitivity	Specificity	PPV	NPV
Caprini risk assessment model	0.497	0.599	>9	0.265	0.875	0.775	0.423

AUC = Area Under Curve, PPV=Positive Predictive Value, NPV=Negative Predictive Value.

## 4 Discussion

In this study, six key risk factors linked to the occurrence of VTE in lung transplant recipients were uncovered. These factors interacted in complex ways and collectively drove the development and progression of VTE. The SHAP algorithm was used to provide visual elucidations of the model. The global interpretation quantified the relative significance of each feature in contributing to VTE risk, whereas the local interpretation highlighted how individual features specifically influenced the prediction for each sample. These SHAP explanations enhanced the interpretability and transparency of the model, thereby bolstering its clinical applicability.

The traditional CRS is widely used in clinical practice to assess the risk of VTE (14). However, the CRS has certain limitations, such as the inclusion of too many variables and some non-routine testing items. The ML model presented in this study may offer advantages in predicting VTE. Our research identified several critical risk factors for VTE in lung transplantation, including the use of anticoagulants. Generally, anticoagulants are administered to prevent thrombus formation. Risk assessment using the Padua or Caprini model is standard practice, with high-risk patients receiving prophylactic anticoagulation therapy with low-molecular-weight heparin (15). Clinically, the use of anticoagulants requires careful balancing of thromboprophylactic efficacy against the risk of hemorrhage, as suboptimal VTE prophylaxis may result from insufficient consideration of this risk–benefit ratio (16).

High BMI was previously identified as a potential risk factor for VTE (17, 18). Moneke et al. reported that patients with a BMI > 25 are at substantially increased risk of thromboembolic events following lung transplantation (19). Another study indicated that patients with a high BMI (≥30 kg/m^2^) were at greater risk of experiencing thromboembolic events after lung transplantation (20). The increased adipose tissue in obese patients may elevate blood viscosity, thereby increasing the risk of thrombus formation. Moreover, obesity can lead to excessive inflammatory responses, further promoting thrombogenesis (21).

A growing body of evidence supports the use of procalcitonin for bacterial infections and to guide antibiotic therapy (21). For lung transplant patients on ECMO, procalcitonin levels indirectly reflect systemic inflammation and infection. Infection and inflammation can lead to a hypercoagulable state, thereby increasing the risk of thrombus formation (22). Therefore, monitoring procalcitonin levels, in conjunction with other biomarkers and clinical assessments, may aid in the effective prevention and management of VTE risk in lung transplant patients during ECMO support.

Our study identified a history of thrombosis as one of the major risk factors for VTE. A history of thrombosis revealed the presence of hereditary or acquired thrombophilia in patients, which increases the risk of thrombus formation (23). Lung transplantation is a high-risk procedure and during ECMO support, localized hemodynamic changes may occur, which can lead to a hypercoagulable state. Patients with a history of thrombosis are more likely to enter this hypercoagulable state postoperatively, thereby increasing the probability of VTE (24). In patients with a history of thrombosis, this risk may be further amplified with the use of immunosuppressive agents.

Concurrent respiratory failure was identified as a critical risk factor for VTE in lung transplant recipients undergoing ECMO. Patients with respiratory failure often have multiple underlying diseases, which can lead to impaired fibrinolysis and thereby increased risk of thrombus formation (25). Furthermore, elevation of white blood cell count is closely associated with an increased risk of venous thrombus formation. However, white blood cell count does not serve as the exclusive determinant, as its role may involve interactions with other inflammatory markers and pathological mechanisms (26). Further studies are needed to explore the specific implications of white blood cell count in different disease contexts and its application value in the prevention and treatment of VTE.

This study used ML methods to develop a model to predict the risk of VTE in lung transplant recipients. Six different ML algorithms (RF, LR, XGBoost, SVM, KNN, and AdaBoost) were used to construct predictive models. The RF model demonstrated the highest efficacy in predicting VTE risk in lung transplant patients. This superior performance may be attributed to the RF model’s bagging-based architecture with randomized feature selection, which reduces variance and overfitting more effectively than the boosting strategies employed by XGBoost and AdaBoost, particularly in the context of limited-sample clinical data with inherent noise. This approach effectively handles multidimensional features and potential complex relationships while reducing the risk of overfitting. Additionally, the RF model exhibits robustness against noise and outliers, enhancing its stability and reliability when processing real-world data.

This study has several limitations. First, the decline in AUC from the training set (0.990) to the validation set (0.895) indicates mild overfitting, likely due to the limited sample size (n = 189) and high feature-to-observation ratio. Despite using RFE for dimensionality reduction and SMOTE for class imbalance, the model may have captured training-specific patterns. Future multicenter studies with larger cohorts are warranted to confirm the model’s generalizability. Second, while we identified several key risk factors, unconsidered potential variables or confounding factors might exist, as our analysis did not exhaustively account for all possible determinants. Third, the validation was performed using a temporal validation approach, utilizing data from the same institution but from a later time period, rather than true external validation involving multicenter, geographically diverse cohorts. This may limit the generalizability of our findings. Future studies should conduct rigorous external validation across multiple centers and regions to confirm the robustness and portability of this model.

## 5 Conclusion

This study developed a machine learning-based predictive model for VTE in patients undergoing lung transplantation with ECMO support. Using a large dataset of ECMO-supported lung transplantation cases, we identified clinical indicators strongly correlated with thrombus formation and developed a predictive model to improve the accuracy and timeliness of VTE risk prediction. For each patient, we dynamically adjusted thrombosis risk assessments to inform personalized anticoagulation strategies. These research findings may be applied to clinical practice to optimize thromboprophylaxis protocols in ECMO-supported patients, thereby improving outcomes for lung transplant recipients and enhancing healthcare quality and efficiency.

## Data Availability

The raw data supporting the conclusions of this article will be made available by the authors, without undue reservation.
