# Supplementary material for: Construction and validation of a machine learning-based prediction model for venous thromboembolism in lung transplant recipients supported by ECMO
Source: Front Med (Lausanne). 2026 Jun 4;13:1808657. doi: 10.3389/fmed.2026.1808657 (PMC13275708; doi:10.3389/fmed.2026.1808657)
Supplement: Supplementary file 1 [file Supplementary_file_1.docx]

**Supplementary Material S1**

**Hyperparameter Tuning Configuration**

for Machine Learning Algorithms

**Overview**

This document provides a complete description of the hyperparameter search space for each machine learning algorithm employed in this study. All models were optimized using GridSearchCV with 5-fold cross-validation and accuracy as the scoring metric. The best model was selected via grid_search.best_estimator_.

**1. AdaBoost Classifier**

| **Parameter** | **Search Space** | **Description** |
| --- | --- | --- |
| **n_estimators** | [50, 100, 150] | Number of weak learners |
| **learning_rate** | [0.01, 0.1, 1] | Learning rate (shrinkage factor) |

*Base model: AdaBoostClassifier(n_estimators=50, random_state=42)*

**2. K-Nearest Neighbors (KNN)**

| **Parameter** | **Search Space** | **Description** |
| --- | --- | --- |
| **n_neighbors** | [3, 5, 7, 9] | Number of neighbors |
| **weights** | ['uniform', 'distance'] | Weight function (uniform or distance-based) |
| **p** | [1, 2] | Distance metric (1: Manhattan, 2: Euclidean) |

*Base model: KNeighborsClassifier()*

**3. Logistic Regression**

| **Parameter** | **Search Space** | **Description** |
| --- | --- | --- |
| **C** | [0.001, 0.01, 0.1, 1, 10, 100] | Inverse of regularization strength |
| **penalty** | ['l1', 'l2'] | Regularization type (L1 or L2 norm) |
| **solver** | ['liblinear', 'lbfgs'] | Optimization algorithm |

*Base model: LogisticRegression(max_iter=1000)*

**4. Support Vector Machine (SVM)**

| **Parameter** | **Search Space** | **Description** |
| --- | --- | --- |
| **C** | [0.1, 1, 10, 100] | Regularization parameter |
| **kernel** | ['linear', 'rbf', 'poly'] | Kernel function type |
| **gamma** | ['scale', 'auto'] | Kernel coefficient |

*Base model: SVC(probability=True, max_iter=1000)*

**5. Random Forest**

| **Parameter** | **Search Space** | **Description** |
| --- | --- | --- |
| **n_estimators** | [100, 200, 300] | Number of trees |
| **max_depth** | [None, 10, 20, 30] | Maximum depth of the tree |
| **min_samples_split** | [2, 5, 10] | Minimum samples to split a node |
| **min_samples_leaf** | [1, 2, 4] | Minimum samples at a leaf node |
| **bootstrap** | [True, False] | Whether to use bootstrap sampling |

*Base model: RandomForestClassifier(random_state=42)*

**6. XGBoost**

| **Parameter** | **Search Space** | **Description** |
| --- | --- | --- |
| **n_estimators** | [100, 200] | Number of decision trees |
| **learning_rate** | [0.01, 0.1] | Learning rate (shrinkage) |
| **max_depth** | [3, 6] | Maximum depth of the tree |
| **subsample** | [0.8, 1] | Subsample ratio of training instances |
| **colsample_bytree** | [0.8, 1] | Subsample ratio of columns per tree |

*Base model: XGBClassifier(use_label_encoder=False, eval_metric='logloss')*

**Common Configuration Summary**

| **Setting** | **Value** |
| --- | --- |
| **Search Method** | Grid Search (exhaustive) |
| **Cross-Validation** | 5-fold CV |
| **Scoring Metric** | Accuracy |
| **Parallel Jobs** | n_jobs = -1 (all CPU cores) |
