# Supplementary material for: Construction and validation of a machine learning-based prediction model for venous thromboembolism in lung transplant recipients supported by ECMO
Source: Front Med (Lausanne). 2026 Jun 4;13:1808657. doi: 10.3389/fmed.2026.1808657 (PMC13275708; doi:10.3389/fmed.2026.1808657)
Supplement: Supplementary file 2 [file Supplementary_file_2.docx]

**Supplementary Material S2**

**Mathematical Equations for Comparison Metrics**

This supplementary material presents the mathematical definitions for the seven comparison metrics used to evaluate the performance of machine learning models in this study. Let TP, TN, FP, and FN denote true positives, true negatives, false positives, and false negatives, respectively, as defined by the confusion matrix.

**Supplementary Equation 1. Accuracy**

Accuracy measures the proportion of correctly classified instances among all instances:

$$Accuracy = \frac{TP + TN}{TP + TN + FP + FN}$$

**Supplementary Equation 2. Sensitivity (Recall)**

Sensitivity (also known as recall or true positive rate) measures the proportion of actual positives correctly identified:

$$Sensitivity = \frac{TP}{TP + FN}$$

**Supplementary Equation 3. Specificity**

Specificity (also known as true negative rate) measures the proportion of actual negatives correctly identified:

$$Specificity = \frac{TN}{TN + FP}$$

**Supplementary Equation 4. Positive Predictive Value (PPV)**

PPV (also known as precision) measures the proportion of positive predictions that are actually correct:

$$PPV = \frac{TP}{TP + FP}$$

**Supplementary Equation 5. Negative Predictive Value (NPV)**

NPV measures the proportion of negative predictions that are actually correct:

$$NPV = \frac{TN}{TN + FN}$$

**Supplementary Equation 6. F1 Score**

The F1 score is the harmonic mean of precision (PPV) and recall (sensitivity), providing a balanced measure of model performance:

$$F_{1} = 2 \times\frac{PPV \times Sensitivity}{PPV + Sensitivity}$$

**Supplementary Equation 7. Area Under the ROC Curve (AUC)**

AUC represents the area under the receiver operating characteristic curve, where TPR(t) is the true positive rate and FPR(t) is the false positive rate at classification threshold t:

$$AUC = \int_{0}^{1} TPR(t) d t$$
