# Supplementary material for: Construction and validation of a machine learning-based prediction model for venous thromboembolism in lung transplant recipients supported by ECMO
Source: Front Med (Lausanne). 2026 Jun 4;13:1808657. doi: 10.3389/fmed.2026.1808657 (PMC13275708; doi:10.3389/fmed.2026.1808657)
Supplement: Supplementary file 3 [file Table_1.docx]

**Supplementary Table 1 Number of variables and corresponding accuracy**

| Number of Variables | Kappa | Accuracy | Selected |
| --- | --- | --- | --- |
| 6 | 0.886 | 0.763 | * |
| 8 | 0.877 | 0.742 |  |
| 10 | 0.880 | 0.748 |  |
| 22 | 0.881 | 0.749 |  |
